# Substance abuse among high school learners in a rural education district in the Free State province, South Africa

**DOI:** 10.4102/safp.v63i1.5302

**Published:** 2021-08-23

**Authors:** Kebogile E. Mokwena, Nomkanka J. Setshego

**Affiliations:** 1 Department of Public Health, Sefako Makgatho Health Sciences University, Pretoria, South Africa

**Keywords:** substance abuse, high school, rural area, Free State province, South Africa

## Abstract

**Background:**

In South Africa, many studies conducted on substance abuse among in-school youth focus on urban areas. However, anecdotal evidence suggests that rural areas are experiencing an increase in substance abuse, though there is dearth of studies in these areas.

**Methods:**

This study used a quantitative design to collect data from 629 high school learners who were in Grades 10 and 11 in public schools in rural Free State Province, to determine the prevalence of, and factors associated with substance use.

**Results:**

The sample consisted of 46% males and 54% females. Their ages ranged from 14 to 20 years, with a mean of 16.9 years. The prevalence of substance abuse was 47% (*n* = 295) with alcohol consumption, cigarette and dagga smoking being the most common substances used. Socio-demographically, age and gender were significantly associated with substance abuse. While behavioural variables of physical fights, serious problems with parents and friends, poor academic performance, trouble with police, having sex without condom, and having sex and regretted the next day were significantly associated with substance abuse (*p* = 0.05).

**Conclusion:**

The prevalence of substance abuse is very high for this rural school community, which highlights the need to pay attention to rural schools regarding substance abuse challenges.

## Introduction

South Africa has a high rate of substance abuse among young people, which includes both in and out of school youth. Despite reported stigma and associated reluctance to seek treatment for substance abuse,^1^ an increase in young people aged 20 years seeking treatment for substance abuse,^2^ is an indication of the gravity of the problem. Variations in the trends of substance use have been reported between urban and rural youth,^3^ with substance use among learners in urban area being more than those in rural areas.^4^ However, rural areas are increasingly experiencing problems of adolescent substance use.^5^

Substance abuse among learners is associated with a range of criminal acts,^6,7^ which includes violence and bullying.^8,9^ It has also been associated with a range of mental disorders^10^; while socially, it is associated with social disorganisation, deviant behaviour, and social interaction with deviant groups,^11^ depending on which substances are used. Risky sexual behaviours are also likely to manifest into sexually transmitted infections.

In the school environment and academic context, substance abuse has been associated with challenges in school discipline,^12^ appetitive aggression^7^ and other classroom management challenges.^13^ These frustrate the achievement of intended education outcomes, and result in poor academic performance, including possible dropping out of school.

The social environment is often a significant determinant of substance abuse,^14^ with adolescents being often influenced by their peers.^15^ Other determinants of drug use among young people include: curiosity, sense of growing up, amount or lack of parental discipline and monitoring, and family cohesion.^16^ Availability and easy access of illicit drugs within the community or the household,^17^ economic hardship, high unemployment, lack of adequate social support networks, pressure to meet daily family needs, family conflicts,^18^ were all associated with use of illicit drugs.

In the context of schooling, substance abuse has been significantly associated with poor academic performance,^19^ which often results in dropping out of school.^20,21,22,23,24^ The perceptions that rural areas experience less substance abuse among learners often result in inadequate attention being paid to such areas. With the precise intention of expanding substance abuse studies to areas other than cities, the purpose of this study was to determine the prevalence of substance abuse, as well as explore the associations between substance abuse and a range of demographic variables among learners in a rural school district of Free State Province, South Africa.

## Methodology

### Study design

A cross-sectional quantitative survey, using a self-developed questionnaire, was conducted among learners attending local high schools in the rural school district of Free State Province, South Africa.

### Study setting

The study was conducted at high schools at Setsoto local municipality, Thabo Mofutsanyane District of the Eastern Free State, which is largely rural. The municipality is comprised of four rural towns namely Clocolan/Hlohlolwane, Marquard/Moemaneng, Senekal/Matwabeng, and Ficksburg/Meqheleng. According to the Census 2011, the municipality has a population of 1 10 335, and of those aged 20 years and above approximately 8.7% have no formal schooling, 22.6% have completed matric, and 6.9% have some form of tertiary education. Agriculture is the main economic activity in the municipality, and the unemployment rate of those aged between 15 and 34 is high at 46%.

### Study population and sample

The study population was high school learners in public schools of Setsoto municipality. There are eight public schools in the sub-district, and using the hat method, four schools from each rural town were randomly selected. An additional school was used for the pilot study. The estimated population of Grades 10 and 11 in the eight schools is 2100.

### Sample and sampling technique

The sample consisted of learners who were in Grades 10 and 11 at the time of data collection. From estimated population size of 2100 from eight schools, the Raosoft sample calculator was used to determine a minimum sample for the study. Using a 5% margin of error, a confidence level of 95% and a distribution of 50%, a minimum sample size of 323 was calculated. Because a survey was used, in which all learners willing to participate in the study were invited, 800 learners in 42 classrooms participated, but 629 were analysed, with the rest having missing information of more than 10%.

### Recruitment

Recruitment was done at the identified school, with the researcher addressing the Grades 10 and 11 learners by telling them about the study and requesting them to participate. Those who agreed to participate were given letters for their parents to provide informed consent.

### Data collection tool

An English self-administered questionnaire, which was modified from a risk behaviour survey, was used to collect data. The tool was pilot tested among 20 learners at another school before data collection. The tool collected learner-related demographic data like age, gender, grade, and whether they have ever repeated a class, as well as the socio-economic data of the family, such as employment status of parents, highest education attained by the parents and who the participants live with. Substance use related data collected included the substances of current use, age at which they first experimented with substances, use of substances in their social environment and ease of access of substances. Behaviour related data included whether they were involved in physical fights, were in trouble with police, were engaged in risky sexual behaviour, (such as having sex without using a condom or having sex and regretted it the next day), and problems with parents and friends.

### Data collection

On the day of data collection, learners whose parents had provided the informed consent were assembled in the school hall or classroom and an explanation about the study was repeated. The learners were given an opportunity to ask questions or seek clarification. Informed consent was administered to learners who were over the age of 18, while learners under the age of 18, whose parents had provided consent, were requested to provide assent by signing the appropriate forms. The data collection tool was then distributed to all the learners. Adequate time was given to complete the questionnaires, and the learners left the venue after all had completed the process.

### Data analysis

The data were captured into Microsoft Excel and transported to STATA version 13 for analysis. Descriptive statistics were used to analyse socio-demographic data prevalence of substance use, and these were reflected in the form of frequencies and percentages. Chi-square test was used to explore associations between a range of demographical variables and substance abuse among the sample. Statistical significance was set at ≤ 0.05.

### Validity, reliable and bias

At sub-district district level, selection bias was minimised by random selection of schools. The use of the survey at school minimised selection bias as all learners in the selected grades who were prepared to participate were included in the study. The questionnaire was pilot tested to identify any challenges before the actual data collection commenced.

### Ethical considerations

Ethical approval for the study was obtained from the Sefako Makgatho Health Sciences University Research and Ethics Committee (number: SMUREC/H/95/2016). Permissions to conduct the study were obtained from the offices of the Provincial Department of Education, the Thabo Mofutsanyane District of Education, the Setsoto sub-district and the management of each participating school. Informed consent was obtained from parents for participants who were younger than 18 years of age, and these minors provided assent to participate in the study. Informed consent was obtained from participants who were 18 years and above.

## Results

### Characteristics of the sample

Eight hundred (800) learners participated in the survey, and of these, 629 were analysed, with the others being excluded because of missing data of 10% or more. Of the 629 students whose data were analysed, more than half (55%) were in the age group 16–17 years followed by those aged 18 years and above (33%). Their ages ranged from 14 to 20 years, with a mean of 16.9.

### Prevalence and type of substance use among learners

The prevalence of substance use among the study participants was 47%, and of those using substances, the highest proportion consume alcohol (87%) followed by cigarette (45%) and (24%) dagga smoking (Table 1).

**TABLE 1:** Prevalence and types of substance use among learners (*n* = 295).

Substance	No	%
Alcohol	256	87
Cigarette	134	45
Dagga	72	24
Nyaope	11	4
Cocaine	7	2
Ecstasy	6	2
Other	18	6

Note: The total exceeds 100% because some participants reported more than one substance.

### Factors associated with substance use

The association between substance use and selected demographic characteristics are shown in Table 2. Substance use significantly increased with age (*p* < 0.05), while the prevalence was significantly higher among males than females (53% vs. 42%, *p* < 0.05). A significant higher proportion of the participants were introduced to substance use by friends (*p* < 0.05). There was no statistical association between substance use and grades, employment status of the parent, and whether they stay with their parents or not.

**TABLE 2:** Association between demographics and substance use.

Variables	Total	Non-user		User		*p*
		*N*	%	*N*	%	
**Age in years**						
≤ 15	78	51	65	27	35	0.002
16–17	346	193	56	153	44	
≥ 18	205	90	44	115	56	
**Gender**						
Male	288	136	47	152	53	0.007
Female	341	198	58	143	42	
**Grades**						
10	459	252	55	207	45	0.137
11	170	82	48	88	52	
**Employment status of parents**						
Both employed	107	53	49	54	51	0.560
Mother employed	179	90	50	89	50	
Father employed	163	89	55	74	45	
Both unemployed	179	101	56	78	44	
**Stay with**						
Alone	24	17	71	7	29	0.107
Both parents	221	112	51	109	49	
Father	36	14	39	22	61	
Mother	194	112	58	82	42	
Grand parents	134	67	50	67	50	
Family	20	12	60	8	40	
**Who introduced them to substances**						
Parent	9	3	33	6	67	< 0.001
Friends	261	67	26	194	74	
Sibling	27	10	37	17	63	
Not stated	310	236	76	74	24	

Table 3 illustrates behaviours that were significantly associated with substance use.

**TABLE 3:** Associations between behaviours and substance use.

Behaviour	Non-user		User		*p*
	*n*	%	*n*	%	
Physical fights	75	23	122	42	< 0.001
Accident or injury	68	21	78	27	0.070
Serious problems with parents	87	26	121	41	< 0.001
Serious problems with friends	128	37	154	53	< 0.001
Performed poorly at school	149	45	175	60	< 0.001
Being a victim of robbery	28	8	37	13	0.078
Trouble with police	13	4	32	11	< 0.001
Had sex without condom	79	24	107	37	< 0.001
Had sex and regretted the next day	62	19	89	31	< 0.001

## Discussion

The purpose of the study is to determine the prevalence of substance abuse, as well as explore the association between substance abuse and a range of demographic variables. The prevalence of substance abuse among this sample is high at 47%, which is close to the 47.9% reported in a similar sample in Ethiopia,^25^ but higher than the 6% reported in another study conducted in a rural setting in South Africa.^4^ The finding that many learners started using substances at a young age of 15 years confirms findings of a previous study conducted in South Africa.^16^ Although cigarettes and alcohol are legal, they are still illegal for minors such as most of the sample. Of greater concern is the use of illicit drugs such as dagga, nyaope, ecstasy and cocaine, which indicates criminality as these are prohibited substances. Dagga is easily cultivated and commonly used in South Africa, and nyaope is a cocktail drug that has destroyed many lives among Black communities in South Africa because of its high addictive characteristics.^26,27,28,29,30^

Adolescents who use drugs have been reported to have significantly lower levels of psychological well-being and life satisfaction,^31,32^ which implies mental and social risks for the sample. Although peer pressure may influence young people to use drugs, they still feel guilty and stigmatised by family and community, which increases the chances of social ill-health,^33^ which increases the shame associated with the behaviour of using substance.^34^ The challenge of substance abuse should therefore be understood comprehensively as a problem of adolescent social ill-health.

Males had a significant higher prevalence of substance use than females (*p* = 0.007), which is similar to previous studies which reported that males were up to 10 times more likely to use substances than females.^35,36^ The finding that older learners are more likely to use substances than younger ones is similar to a study conducted among learners in the Western Cape, which reported the odds ratio of 1.6 among older learners.^36^ Also similar is the finding that substance use by other members of the household and friends, increases the risk of use among learners two-fold.^36^ These findings highlight the need for comprehensive interventions to influence the comprehensive well-being of young people, especially among young learners. Such interventions also need a community component,^37,38^ which is likely to improve the effectiveness of substance use prevention amongst learners, and thus improve the overall well-being of these young people.

Substance use has been shown to be associated with poor academic performance,^39,40,41^ a serious barrier to reaching the goals of the education system. The finding of a significant association between substance use and risky sexual behaviour^42^ confirms the negative impact of substance abuse on overall youth health.

The significant association of substance abuse with a range of anti-social behaviours of physical fights, serious problems with parents and friends, poor academic performance, trouble with police, having sex without condom and having sex and regretted the next day, all with *p*-values of 0.001, are similar to the findings reported in previous studies, which reported statistically significant associations ranging from *p*-values of 0.001–0.05.^36,43^ These findings put substance abuse at the centre of various problems experienced at South African schools and communities. These associations also identify the need to target substance abuse as a barrier to overall social development because the outcomes, be they academic, physical health, social and/or mental have long-term implications for the affected learners.

Alcohol, cannabis and cigarettes were found to be the most commonly used substances, which is similar to another study conducted in Durban, South Africa.^44^ The ease of access for these substances increases the levels of challenges as this cannot be addressed without the involvement of other sectors, including the law enforcement and behavioural scientists. The complexity of the situation also indicates the urgency of stakeholders to work together to develop interventions that are focused on both prevention and management. Currently, there are no such interventions accessible by the general learner in any South African public-school setting.

Despite the challenges of substance abuse in schools, it does not seem that the Department of Education has any specific intervention to address the problem, other than relying on the Life Orientation (LO) learning area, which aims to address a wide range of learner developmental areas. which include personal, psychological, neuro-cognitive, motor, physical, moral, spiritual, cultural and socio-economic areas.^45^ However, the delivery of LO has been reported to have serious challenges because of constraints at the individual, interpersonal, school, and community levels.^46^ Another shortcoming of LO as a resource for substance abuse is that it is general, and does not address personal experiences,^47^ and therefore is limited in assisting learners with substance abuse challenges. Of serious concern is that the Department of Basic Education’s policy on management of substance abuse in schools is neither known nor implemented,^48^ which implies that there is not much at school level that addresses the serious problem of substance abuse among learners.

### Limitations of the study

As with other survey studies, 21% of the questionnaires could not be analysed because of missing data. However, the high response rate of 78% and a relatively large sample size of 629 counteracted the non-usable questionnaires. A limitation which applies to other survey studies is the response bias, in which the sample may under- or over-estimate the population parameter. However, this potential bias was minimised by sampling from various schools and more than one class in a school. Societal lack of approval of substance abuse by learners may have contributed to bias in their responses, but this was minimised by the privacy afforded to the participants, which meant that others would not have known about their responses.

## Conclusion

The result of the study contributes to highlight the need for interventions to address the challenge of substance abuse in schools, which will improve the academic outcomes with long-term social and career impacts. As substance abuse is more of a societal rather than just a school’s challenge, the required interventions need not be limited to schools, but extend to other young people in communities, including rural areas.

### Recommendations

It is recommended that the substance abuse problem be outsourced to public health and/or behavioural health specialist and not be left to the Department of Education, as this is not their focus areas. This will enable consistent application, monitoring and evaluation of such interventions, and enhance the implementation of necessary modifications.
